# (4-Chlorobenzoyl)(4-chlorophenyl)amino 3-(2-nitrophenyl)propanoate

**DOI:** 10.1107/S1600536813007174

**Published:** 2013-03-23

**Authors:** Yi-lan Ding, Chang-jiang Shao

**Affiliations:** a Lanzhou Maternal and Child Health Care Hospital, Lanzhou 730000, Gansu Province, People’s Republic of China; b The Peoples First Hospital of Lanzhou, Lanzhou 730000, Gansu Province, People’s Republic of China

## Abstract

In the title hydroxamic acid derivate, C_22_H_16_Cl_2_N_2_O_5_, the nitro-substituted benzene ring forms dihedral angles of 14.11 (15) and 16.08 (15)°, with the 4-chloro­benzoyl and 4-chloro­phenyl benzene rings, respectively. The dihedral angle between the chloro-substituted benzene rings is 2.28 (13)°. In the crystal, mol­ecules are linked by weak C—H⋯O hydrogen bonds, forming chains along [100].

## Related literature

For applications of hydroxamic acid derivatives, see: Noh *et al.* (2009 ▶); Zeng *et al.* (2003 ▶). For the synthesis, see: Ayyangark *et al.* (1986 ▶). For related structures, see: Zhang *et al.* (2012 ▶); Ma *et al.* (2012 ▶).
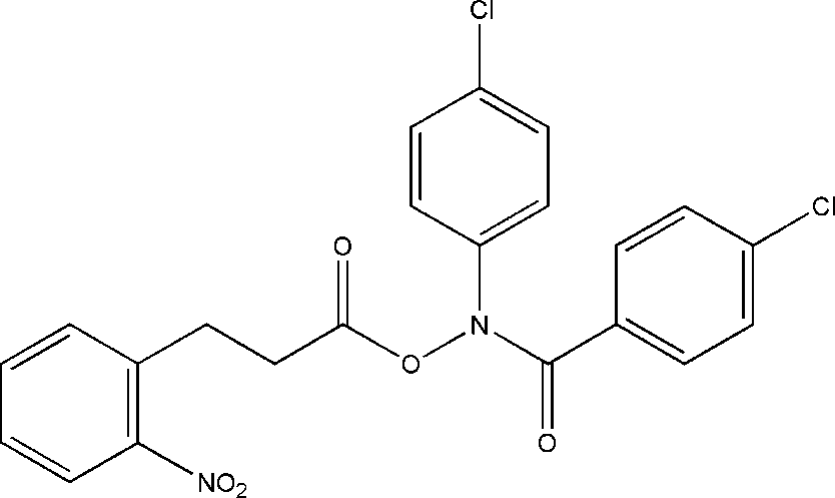


## Experimental

### 

#### Crystal data

C_22_H_16_Cl_2_N_2_O_5_
*M* *_r_* = 459.27
Triclinic, 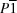

*a* = 6.1710 (3) Å
*b* = 12.8881 (7) Å
*c* = 13.3490 (8) Å
α = 89.933 (5)°
β = 76.959 (5)°
γ = 82.114 (4)°
*V* = 1024.03 (10) Å^3^
*Z* = 2
Mo *K*α radiation
μ = 0.36 mm^−1^
*T* = 294 K
0.32 × 0.28 × 0.25 mm

#### Data collection

Agilent SuperNova (Dual, Cu at zero) Eos diffractometer
Absorption correction: multi-scan (*CrysAlis PRO*; Agilent, 2011 ▶) *T*_min_ = 0.757, *T*_max_ = 1.000
7506 measured reflections
4578 independent reflections
3269 reflections with *I* > 2σ(*I*)
*R*_int_ = 0.021

#### Refinement

*R*[*F* ^2^ > 2σ(*F* ^2^)] = 0.044
*wR*(*F* ^2^) = 0.113
*S* = 1.02
4578 reflections
280 parameters
H-atom parameters constrained
Δρ_max_ = 0.17 e Å^−3^
Δρ_min_ = −0.28 e Å^−3^

### 

Data collection: *CrysAlis PRO* (Agilent, 2011 ▶); cell refinement: *CrysAlis PRO*; data reduction: *CrysAlis PRO*; program(s) used to solve structure: *SUPERFLIP* (Palatinus & Chapuis, 2007 ▶); program(s) used to refine structure: *SHELXL97* (Sheldrick, 2008 ▶); molecular graphics: *OLEX2* (Dolomanov *et al.*, 2009 ▶); software used to prepare material for publication: *OLEX2*.

## Supplementary Material

Crystal structure: contains datablock(s) global, I. DOI: 10.1107/S1600536813007174/lh5593sup1.cif

Structure factors: contains datablock(s) I. DOI: 10.1107/S1600536813007174/lh5593Isup2.hkl

Supplementary material file. DOI: 10.1107/S1600536813007174/lh5593Isup3.cml

Additional supplementary materials:  crystallographic information; 3D view; checkCIF report

## Figures and Tables

**Table 1:** Hydrogen-bond geometry (Å, °)

*D*—H⋯*A*	*D*—H	H⋯*A*	*D*⋯*A*	*D*—H⋯*A*
C4—H4⋯O1^i^	0.93	2.40	3.177 (2)	140
C17—H17*B*⋯O4^ii^	0.97	2.55	3.515 (3)	171

Symmetry codes: (i) 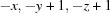; (ii) 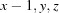.

**Fig. 1.**
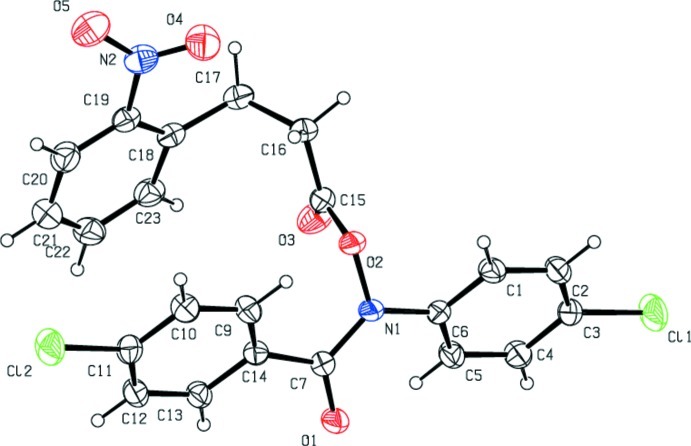
The molecular structure of the title compound with 30% probability displacement dllipsoids. H atoms are shown as small spheres of arbitrary radius.

## supplementary crystallographic information

### Comment

Hydroxamic acid derivatives have received considerable attention in recent years as the result of the discovery of their role in the biochemical toxicology of many drugs and other chemicals (Noh *et al.*, 2009; Zeng *et al.*, 2003). We have performed the crystal structure determination of the title hydroxamic acid derivative.

The molecular structure of the title compound is shown in Fig. 1. The nitro-substituted benzene ring (C18-C23) forms dihedral angles of 14.11 (15) and 16.08 (15)°, with the p-chloro (C9-C14) and p-chloro-substituted (C1-C6) benzene rings, respectively. The dihedral angle between the two chloro-substituted benzene rings is 2.28 (13) °. In the crystal, molecules are linked by weak C—H···O hydrogen bonds to form chains along [100]. Closely related structures appear in the literature (Zhang *et al.*, 2012; Ma *et al.*, 2012).

### Experimental

The title compound (I) was prepared according to the method described by Ayyangark *et al.* (1986). Crystals of (I) suitable for single-crystal X-ray analysis were grown by slow evaporation of a solution of (I) in dichloromethane-methanol (1:3 *v*/*v*).

### Refinement

Hydrogen atoms were placed in calculated positions with C—H = 0.93 and 0.97Å and included in a riding-model approximation with U_iso_(H) = 1.2U_eq_(C).

### Crystal data

**Table:**

C_22_H_16_Cl_2_N_2_O_5_	*Z* = 2
*M**_r_* = 459.27	*F*(000) = 472
Triclinic, *P*1	*D*_x_ = 1.489 Mg m^−^^3^
*a* = 6.1710 (3) Å	Mo *K*α radiation, λ = 0.7107 Å
*b* = 12.8881 (7) Å	Cell parameters from 2661 reflections
*c* = 13.3490 (8) Å	θ = 3.1–28.4°
α = 89.933 (5)°	µ = 0.36 mm^−^^1^
β = 76.959 (5)°	*T* = 294 K
γ = 82.114 (4)°	Block, colourless
*V* = 1024.03 (10) Å^3^	0.32 × 0.28 × 0.25 mm

### Data collection

**Table:**

Agilent SuperNova (Dual, Cu at zero) Eos diffractometer	4578 independent reflections
Radiation source: SuperNova (Mo) X-ray Source	3269 reflections with *I* > 2σ(*I*)
Mirror monochromator	*R*_int_ = 0.021
Detector resolution: 16.0733 pixels mm^-1^	θ_max_ = 28.5°, θ_min_ = 3.1°
ω scans	*h* = −8→8
Absorption correction: multi-scan (*CrysAlis PRO*; Agilent, 2011)	*k* = −16→16
*T*_min_ = 0.757, *T*_max_ = 1.000	*l* = −17→14
7506 measured reflections	

### Refinement

**Table:**

Refinement on *F*^2^	0 restraints
Least-squares matrix: full	H-atom parameters constrained
*R*[*F*^2^ > 2σ(*F*^2^)] = 0.044	*w* = 1/[σ^2^(*F*_o_^2^) + (0.0388*P*)^2^ + 0.2287*P*] where *P* = (*F*_o_^2^ + 2*F*_c_^2^)/3
*wR*(*F*^2^) = 0.113	(Δ/σ)_max_ < 0.001
*S* = 1.02	Δρ_max_ = 0.17 e Å^−^^3^
4578 reflections	Δρ_min_ = −0.28 e Å^−^^3^
280 parameters	

### Special details

**Table:**

Geometry. All esds (except the esd in the dihedral angle between two l.s. planes) are estimated using the full covariance matrix. The cell esds are taken into account individually in the estimation of esds in distances, angles and torsion angles; correlations between esds in cell parameters are only used when they are defined by crystal symmetry. An approximate (isotropic) treatment of cell esds is used for estimating esds involving l.s. planes.
Refinement. Refinement of F^2^ against ALL reflections. The weighted R-factor wR and goodness of fit S are based on F^2^, conventional R-factors R are based on F, with F set to zero for negative F^2^. The threshold expression of F^2^ > 2sigma(F^2^) is used only for calculating R-factors(gt) etc. and is not relevant to the choice of reflections for refinement. R-factors based on F^2^ are statistically about twice as large as those based on F, and R- factors based on ALL data will be even larger.

### Fractional atomic coordinates and isotropic or equivalent isotropic displacement parameters (Å^2^)

**Table:**

	*x*	*y*	*z*	*U*_iso_*/*U*_eq_	
Cl1	−0.18649 (10)	0.33888 (6)	0.87407 (5)	0.0768 (2)	
Cl2	1.31097 (10)	0.36534 (5)	0.04403 (5)	0.0728 (2)	
O1	0.3913 (2)	0.46847 (10)	0.41480 (11)	0.0514 (4)	
O2	0.6701 (2)	0.23063 (9)	0.47616 (10)	0.0446 (3)	
O3	0.4204 (3)	0.15481 (12)	0.41153 (13)	0.0669 (5)	
O4	1.2426 (3)	−0.06198 (14)	0.30600 (14)	0.0745 (5)	
O5	1.4243 (3)	−0.09770 (15)	0.14952 (16)	0.0870 (6)	
N1	0.5125 (3)	0.32286 (12)	0.48837 (13)	0.0488 (4)	
N2	1.2639 (3)	−0.05271 (14)	0.21312 (17)	0.0567 (5)	
C1	0.3969 (3)	0.28616 (15)	0.66805 (16)	0.0467 (5)	
H1	0.5433	0.2555	0.6661	0.056*	
C2	0.2353 (3)	0.28899 (16)	0.75827 (16)	0.0500 (5)	
H2	0.2719	0.2603	0.8173	0.060*	
C3	0.0195 (3)	0.33459 (15)	0.76041 (15)	0.0451 (5)	
C4	−0.0374 (3)	0.37795 (15)	0.67448 (16)	0.0485 (5)	
H4	−0.1837	0.4093	0.6772	0.058*	
C5	0.1242 (3)	0.37473 (15)	0.58384 (15)	0.0475 (5)	
H5	0.0866	0.4035	0.5250	0.057*	
C6	0.3422 (3)	0.32880 (13)	0.58035 (14)	0.0402 (4)	
C7	0.5349 (3)	0.39283 (14)	0.41068 (14)	0.0408 (4)	
C9	0.9520 (3)	0.33060 (15)	0.32673 (15)	0.0455 (5)	
H9	0.9773	0.3017	0.3877	0.055*	
C10	1.1284 (3)	0.32752 (16)	0.24154 (16)	0.0487 (5)	
H10	1.2719	0.2969	0.2450	0.058*	
C11	1.0896 (3)	0.37022 (16)	0.15145 (16)	0.0475 (5)	
C12	0.8791 (3)	0.41665 (16)	0.14499 (16)	0.0498 (5)	
H12	0.8551	0.4451	0.0837	0.060*	
C13	0.7042 (3)	0.42046 (15)	0.23050 (15)	0.0441 (4)	
H13	0.5621	0.4529	0.2268	0.053*	
C14	0.7361 (3)	0.37675 (13)	0.32217 (14)	0.0390 (4)	
C15	0.5977 (3)	0.14812 (15)	0.43340 (15)	0.0451 (5)	
C16	0.7738 (3)	0.05388 (14)	0.42254 (16)	0.0472 (5)	
H16A	0.7454	0.0137	0.4845	0.057*	
H16B	0.9199	0.0766	0.4152	0.057*	
C17	0.7776 (3)	−0.01623 (15)	0.32980 (16)	0.0479 (5)	
H17A	0.8685	−0.0829	0.3345	0.057*	
H17B	0.6260	−0.0296	0.3314	0.057*	
C18	0.8705 (3)	0.03217 (14)	0.22905 (15)	0.0429 (4)	
C19	1.0947 (3)	0.01729 (15)	0.17520 (16)	0.0450 (5)	
C20	1.1712 (4)	0.06501 (18)	0.08326 (18)	0.0589 (6)	
H20	1.3221	0.0522	0.0498	0.071*	
C21	1.0231 (5)	0.13115 (18)	0.04204 (19)	0.0665 (6)	
H21	1.0729	0.1647	−0.0188	0.080*	
C22	0.8000 (4)	0.14722 (17)	0.09175 (19)	0.0640 (6)	
H22	0.6982	0.1915	0.0639	0.077*	
C23	0.7258 (4)	0.09832 (16)	0.18248 (18)	0.0542 (5)	
H23	0.5735	0.1098	0.2139	0.065*	

### Atomic displacement parameters (Å^2^)

**Table:**

	*U*^11^	*U*^22^	*U*^33^	*U*^12^	*U*^13^	*U*^23^
Cl1	0.0613 (4)	0.0970 (5)	0.0541 (4)	0.0046 (3)	0.0148 (3)	0.0189 (3)
Cl2	0.0545 (3)	0.0921 (5)	0.0570 (4)	0.0001 (3)	0.0116 (3)	0.0122 (3)
O1	0.0516 (8)	0.0480 (8)	0.0467 (8)	0.0080 (6)	−0.0041 (7)	0.0103 (6)
O2	0.0459 (7)	0.0344 (7)	0.0468 (8)	0.0041 (5)	−0.0020 (6)	0.0021 (6)
O3	0.0497 (9)	0.0645 (10)	0.0839 (12)	0.0104 (7)	−0.0210 (9)	−0.0156 (9)
O4	0.0601 (10)	0.0904 (13)	0.0709 (13)	0.0111 (9)	−0.0239 (9)	0.0071 (10)
O5	0.0570 (10)	0.0872 (13)	0.0979 (15)	0.0279 (9)	−0.0008 (10)	−0.0043 (11)
N1	0.0554 (10)	0.0346 (8)	0.0430 (9)	0.0104 (7)	0.0065 (8)	0.0069 (7)
N2	0.0405 (9)	0.0541 (10)	0.0727 (14)	0.0027 (8)	−0.0128 (9)	−0.0007 (10)
C1	0.0417 (10)	0.0455 (10)	0.0475 (12)	0.0044 (8)	−0.0054 (9)	0.0090 (9)
C2	0.0524 (12)	0.0527 (12)	0.0411 (11)	0.0004 (9)	−0.0074 (9)	0.0115 (9)
C3	0.0450 (11)	0.0441 (10)	0.0399 (11)	−0.0027 (8)	0.0009 (9)	0.0033 (8)
C4	0.0391 (10)	0.0528 (12)	0.0491 (12)	0.0025 (9)	−0.0057 (9)	0.0069 (9)
C5	0.0474 (11)	0.0526 (11)	0.0400 (11)	0.0016 (9)	−0.0097 (9)	0.0079 (9)
C6	0.0452 (10)	0.0339 (9)	0.0366 (10)	−0.0018 (8)	−0.0013 (8)	0.0006 (7)
C7	0.0464 (10)	0.0388 (10)	0.0360 (10)	−0.0029 (8)	−0.0087 (8)	0.0018 (8)
C9	0.0461 (11)	0.0508 (11)	0.0412 (11)	−0.0072 (9)	−0.0134 (9)	0.0090 (9)
C10	0.0385 (10)	0.0549 (12)	0.0514 (12)	−0.0048 (9)	−0.0084 (9)	0.0070 (9)
C11	0.0426 (10)	0.0511 (11)	0.0437 (12)	−0.0063 (9)	0.0003 (9)	0.0011 (9)
C12	0.0523 (12)	0.0556 (12)	0.0385 (11)	−0.0022 (9)	−0.0075 (9)	0.0092 (9)
C13	0.0418 (10)	0.0475 (11)	0.0405 (11)	−0.0008 (8)	−0.0076 (9)	0.0061 (8)
C14	0.0425 (10)	0.0362 (9)	0.0384 (10)	−0.0066 (8)	−0.0088 (8)	0.0014 (7)
C15	0.0465 (11)	0.0416 (10)	0.0408 (11)	0.0017 (8)	−0.0016 (9)	0.0032 (8)
C16	0.0497 (11)	0.0379 (10)	0.0496 (12)	0.0045 (8)	−0.0086 (9)	0.0038 (8)
C17	0.0442 (10)	0.0376 (10)	0.0594 (13)	−0.0014 (8)	−0.0093 (10)	−0.0013 (9)
C18	0.0439 (10)	0.0349 (9)	0.0497 (12)	−0.0014 (8)	−0.0126 (9)	−0.0057 (8)
C19	0.0432 (10)	0.0400 (10)	0.0506 (12)	0.0010 (8)	−0.0120 (9)	−0.0043 (9)
C20	0.0560 (13)	0.0579 (13)	0.0571 (14)	−0.0051 (11)	−0.0024 (11)	−0.0039 (11)
C21	0.0851 (18)	0.0564 (13)	0.0553 (15)	−0.0061 (12)	−0.0128 (13)	0.0077 (11)
C22	0.0783 (17)	0.0532 (13)	0.0615 (16)	0.0066 (11)	−0.0276 (14)	0.0049 (11)
C23	0.0488 (11)	0.0522 (12)	0.0604 (14)	0.0063 (9)	−0.0179 (11)	−0.0040 (10)

### Geometric parameters (Å, º)

**Table:**

Cl1—C3	1.7411 (19)	C10—C11	1.378 (3)
Cl2—C11	1.738 (2)	C10—H10	0.9300
O1—C7	1.216 (2)	C11—C12	1.374 (3)
O2—C15	1.379 (2)	C12—C13	1.378 (3)
O2—N1	1.4131 (18)	C12—H12	0.9300
O3—C15	1.187 (2)	C13—C14	1.389 (3)
O4—N2	1.224 (2)	C13—H13	0.9300
O5—N2	1.225 (2)	C15—C16	1.498 (3)
N1—C7	1.369 (2)	C16—C17	1.528 (3)
N1—C6	1.419 (2)	C16—H16A	0.9700
N2—C19	1.463 (3)	C16—H16B	0.9700
C1—C2	1.376 (3)	C17—C18	1.508 (3)
C1—C6	1.383 (3)	C17—H17A	0.9700
C1—H1	0.9300	C17—H17B	0.9700
C2—C3	1.374 (3)	C18—C19	1.395 (3)
C2—H2	0.9300	C18—C23	1.397 (3)
C3—C4	1.370 (3)	C19—C20	1.387 (3)
C4—C5	1.380 (3)	C20—C21	1.370 (3)
C4—H4	0.9300	C20—H20	0.9300
C5—C6	1.384 (3)	C21—C22	1.374 (3)
C5—H5	0.9300	C21—H21	0.9300
C7—C14	1.500 (3)	C22—C23	1.379 (3)
C9—C10	1.382 (3)	C22—H22	0.9300
C9—C14	1.397 (3)	C23—H23	0.9300
C9—H9	0.9300		
			
C15—O2—N1	112.51 (14)	C12—C13—C14	121.23 (18)
C7—N1—O2	117.80 (15)	C12—C13—H13	119.4
C7—N1—C6	127.85 (16)	C14—C13—H13	119.4
O2—N1—C6	114.30 (14)	C13—C14—C9	118.39 (17)
O4—N2—O5	123.0 (2)	C13—C14—C7	115.43 (16)
O4—N2—C19	119.16 (18)	C9—C14—C7	125.93 (17)
O5—N2—C19	117.8 (2)	O3—C15—O2	122.88 (18)
C2—C1—C6	120.22 (18)	O3—C15—C16	127.89 (19)
C2—C1—H1	119.9	O2—C15—C16	109.23 (16)
C6—C1—H1	119.9	C15—C16—C17	112.10 (17)
C3—C2—C1	119.43 (19)	C15—C16—H16A	109.2
C3—C2—H2	120.3	C17—C16—H16A	109.2
C1—C2—H2	120.3	C15—C16—H16B	109.2
C4—C3—C2	121.17 (18)	C17—C16—H16B	109.2
C4—C3—Cl1	118.92 (15)	H16A—C16—H16B	107.9
C2—C3—Cl1	119.91 (16)	C18—C17—C16	112.42 (15)
C3—C4—C5	119.45 (18)	C18—C17—H17A	109.1
C3—C4—H4	120.3	C16—C17—H17A	109.1
C5—C4—H4	120.3	C18—C17—H17B	109.1
C4—C5—C6	120.08 (18)	C16—C17—H17B	109.1
C4—C5—H5	120.0	H17A—C17—H17B	107.9
C6—C5—H5	120.0	C19—C18—C23	114.77 (19)
C1—C6—C5	119.65 (18)	C19—C18—C17	125.79 (18)
C1—C6—N1	118.58 (17)	C23—C18—C17	119.44 (18)
C5—C6—N1	121.76 (17)	C20—C19—C18	123.28 (19)
O1—C7—N1	119.37 (17)	C20—C19—N2	115.92 (19)
O1—C7—C14	120.50 (17)	C18—C19—N2	120.79 (18)
N1—C7—C14	120.13 (16)	C21—C20—C19	119.6 (2)
C10—C9—C14	120.58 (18)	C21—C20—H20	120.2
C10—C9—H9	119.7	C19—C20—H20	120.2
C14—C9—H9	119.7	C20—C21—C22	119.1 (2)
C11—C10—C9	119.39 (18)	C20—C21—H21	120.4
C11—C10—H10	120.3	C22—C21—H21	120.4
C9—C10—H10	120.3	C21—C22—C23	120.6 (2)
C12—C11—C10	121.25 (18)	C21—C22—H22	119.7
C12—C11—Cl2	119.40 (16)	C23—C22—H22	119.7
C10—C11—Cl2	119.35 (16)	C22—C23—C18	122.5 (2)
C11—C12—C13	119.15 (19)	C22—C23—H23	118.7
C11—C12—H12	120.4	C18—C23—H23	118.7
C13—C12—H12	120.4		
			
C15—O2—N1—C7	−92.94 (19)	C10—C9—C14—C13	−0.8 (3)
C15—O2—N1—C6	84.64 (19)	C10—C9—C14—C7	−174.88 (17)
C6—C1—C2—C3	−0.1 (3)	O1—C7—C14—C13	−27.1 (3)
C1—C2—C3—C4	−0.5 (3)	N1—C7—C14—C13	153.69 (17)
C1—C2—C3—Cl1	−179.90 (15)	O1—C7—C14—C9	147.14 (19)
C2—C3—C4—C5	0.7 (3)	N1—C7—C14—C9	−32.1 (3)
Cl1—C3—C4—C5	−179.79 (15)	N1—O2—C15—O3	−1.3 (3)
C3—C4—C5—C6	−0.5 (3)	N1—O2—C15—C16	179.71 (14)
C2—C1—C6—C5	0.2 (3)	O3—C15—C16—C17	32.6 (3)
C2—C1—C6—N1	−178.98 (17)	O2—C15—C16—C17	−148.51 (16)
C4—C5—C6—C1	0.1 (3)	C15—C16—C17—C18	71.5 (2)
C4—C5—C6—N1	179.25 (17)	C16—C17—C18—C19	92.1 (2)
C7—N1—C6—C1	−148.3 (2)	C16—C17—C18—C23	−87.9 (2)
O2—N1—C6—C1	34.4 (2)	C23—C18—C19—C20	0.9 (3)
C7—N1—C6—C5	32.5 (3)	C17—C18—C19—C20	−179.16 (19)
O2—N1—C6—C5	−144.76 (17)	C23—C18—C19—N2	−178.00 (17)
O2—N1—C7—O1	173.77 (16)	C17—C18—C19—N2	2.0 (3)
C6—N1—C7—O1	−3.4 (3)	O4—N2—C19—C20	149.1 (2)
O2—N1—C7—C14	−7.0 (3)	O5—N2—C19—C20	−29.2 (3)
C6—N1—C7—C14	175.80 (17)	O4—N2—C19—C18	−31.9 (3)
C14—C9—C10—C11	−0.2 (3)	O5—N2—C19—C18	149.7 (2)
C9—C10—C11—C12	0.6 (3)	C18—C19—C20—C21	0.6 (3)
C9—C10—C11—Cl2	−179.47 (15)	N2—C19—C20—C21	179.52 (19)
C10—C11—C12—C13	0.1 (3)	C19—C20—C21—C22	−1.3 (3)
Cl2—C11—C12—C13	−179.89 (15)	C20—C21—C22—C23	0.6 (3)
C11—C12—C13—C14	−1.1 (3)	C21—C22—C23—C18	1.0 (3)
C12—C13—C14—C9	1.5 (3)	C19—C18—C23—C22	−1.7 (3)
C12—C13—C14—C7	176.16 (17)	C17—C18—C23—C22	178.38 (19)

### Hydrogen-bond geometry (Å, º)

**Table:**

*D*—H···*A*	*D*—H	H···*A*	*D*···*A*	*D*—H···*A*
C4—H4···O1^i^	0.93	2.40	3.177 (2)	140
C17—H17*B*···O4^ii^	0.97	2.55	3.515 (3)	171

Symmetry codes: (i) −*x*, −*y*+1, −*z*+1; (ii) *x*−1, *y*, *z*.
